# info Hansen: A new online resource for Hansen’s disease

**DOI:** 10.1371/journal.pntd.0008819

**Published:** 2020-10-29

**Authors:** Patrícia Deps

**Affiliations:** Department of Social Medicine and Postgraduate Programme in Infectious Diseases, Federal University of Espírito Santo, Vitória, Espírito Santo, Brazil; Adolfo Lutz Institute of Sao Jose do Rio Preto, BRAZIL

I would like to draw the attention of your readers to a new multilingual online resource on Hansen’s disease, **info Hansen** (www.infohansen.org), which has been created with the help of a team of medical students in Brazil. More than 80% of the 200,000 annual new cases of Hansen’s disease occur in just 3 countries, India, Indonesia, and Brazil, with the latter accounting for >90% of cases in the Latin America [1]. The aim of **info Hansen** is to provide an educational resource created and hosted in Brazil, combining content provided by Brazilian and international contributors on a wide range of topics, that is then disseminated to national and international healthcare professionals, researchers, academics, persons affected by Hansen’s disease, and the public, in Portuguese, Spanish, French, and English.

**info Hansen** arose from an initiative to engage medical students in Brazil with the subject of a disease that remains endemic in their country and that therefore creates a need for newly qualified physicians to have clinical, psychosocial, and historical knowledge that they can use should they encounter Hansen’s disease in their future practice. The site will provide free access to a *Hansen’s disease in Clinical Practice* e-book comprising chapters written by subject matter experts on every aspect of Hansen’s disease, including its history, epidemiology, immunology, environmental sources, clinical manifestations, diagnosis, and treatment. Blog articles will provide an informal means of disseminating novel and thought-provoking scientific, social, and policy ideas about Hansen’s disease.

The involvement of persons affected by Hansen’s disease is vital to the genesis and future development of **info Hansen**. The launch event was promoted by the Brazilian Movement for the Reintegration of Persons Affected by Hansen’s Disease (Movimento de Reintegração das Pessoas Atingidas pela Hanseníase (MORHAN)) [2], with content (blog articles, interviews, and documentary photography) focused on the stigmatisation that is so strongly associated with Hansen’s disease and, sadly, still encountered by persons affected by the disease in their daily lives and still codified in discriminatory legislation in some countries [3]. Several articles on the theme of stigma from Brazil were complemented by articles written by contributors from Europe and Asia, and video interviews with the UN Special Rapporteur on the Elimination of Discrimination against Persons Affected by Hansen’s disease and their Family Members and with persons whose lives were changed by Hansen’s disease. This curation of input from diverse voices and perspectives will be a feature of **info Hansen** as the resource continues to develop and grow. In November, new chapters, videos, and articles will be published. Among them are articles on zoonotic transmission from armadillos, and reflections on the elimination of the disease in Brazil and the world and its obstacles.

Brazil is one of the countries most severely affected by the Coronavirus Disease 2019 (COVID-19) pandemic, and an interprofessional team created info Hansen whilst Universities were closed. On a global health theme is a link between **info Hansen** and a committee established recently to assist migrants from endemic countries such as Brazil who find themselves in non-endemic regions without access to health systems and where Hansen’s disease is a rare occurrence. The Committee to Assist Brazilian Immigrants Afflicted by Hansen’s disease (CAIBAH) will be an electronic sister resource to info Hansen, providing information for immigrants affected by Hansen’s disease who encounter difficulties and prejudice in their new home countries.

**info Hansen** seeks to address the imbalance in information being generated and shared from countries that carry the burden of Hansen’s disease, because Brazil is rich in knowledge, expertise, and human resources. Most importantly, at the heart of **info Hansen** is the recognition of an equal 2-way relationship between health professionals and persons affected by the disease. The flow of information, ideas, and experience across this bridge and the mutual respect that it will engender is essential to the elimination of Hansen’s disease.
